# Recruitment of Antigen Presenting Cells to Skin Draining Lymph Node From HPV16E7-Expressing Skin Requires E7-Rb Interaction

**DOI:** 10.3389/fimmu.2018.02896

**Published:** 2018-12-18

**Authors:** Paula Kuo, Siok Min Teoh, Zewen K. Tuong, Graham R. Leggatt, Stephen R. Mattarollo, Ian H. Frazer

**Affiliations:** Translational Research Institute, The University of Queensland Diamantina Institute, Brisbane, QLD, Australia

**Keywords:** HPV16, CIN, retinoblastoma protein, keratinocytes, APC, skin grafting

## Abstract

“High-risk” human papillomaviruses (HPV) infect keratinocytes of squamous epithelia. The HPV16E7 protein induces epithelial hyperplasia by binding Rb family proteins and disrupting cell cycle termination. Murine skin expressing HPV16E7 as a transgene from a keratin 14 promoter (K14.E7) demonstrates epithelial hyperplasia, dysfunctional antigen presenting cells, ineffective antigen presentation by keratinocytes, and production of immunoregulatory cytokines. Furthermore, grafted K14.E7 skin is not rejected from immunocompetent non-transgenic recipient animals. To establish the contributions of E7, of E7-Rb interaction and of epithelial hyperplasia to altered local skin immunity, K14.E7 skin was compared with skin from K14.E7 mice heterozygous for a mutant Rb unable to bind E7 (K14.E7xRb^ΔL/ΔL^ mice), that have normoplastic epithelium. Previously, we demonstrated that E7-speicfic T cells do not accumulate in K14.E7xRb^ΔL/ΔL^ skin grafts. Here, we further show that K14.E7xRb^ΔL/ΔL^ skin, like K14.E7 skin, is not rejected by immunocompetent non-transgenic animals. There were fewer CD11b^+^ antigen presenting cells in skin draining lymph nodes from animals recipient of K14.E7xRb^ΔL/ΔL^ grafts, when compared with animals receiving K14.E7 grafts or K5mOVA grafts. Maturation of migratory DCs derived from K14.E7xRb^ΔL/ΔL^ grafts found in the draining lymph nodes is significantly lower than that of K14.E7 grafts. Surprisingly, K14.E7xRb^ΔL/ΔL^ keratinocytes, unlike K14.E7 keratinocytes, are susceptible to E7 directed CTL-mediated lysis *in vitro*. We conclude that E7-Rb interaction and its associated epithelial hyperplasia partially contribute to the suppressive local immune responses in area affected by HPV16E7 expression.

## Introduction

Infection of the cervix with high-risk Human Papilloma Virus (HPV) accounts for ~100% of cervical cancer. While most high-risk HPV infections can be cleared spontaneously in immune competent individuals, 1–2% of the infected subjects can progress to cervical intraepithelial neoplasia (CIN), which can persist for decades before developing into cervical cancer (1). A major association between development of cervical premalignancy and specific immune response gene polymorphisms (2) suggests that an inadequate immune response promotes development of premalignancy, but the basis of this inadequate immune response remains uncertain.

HPV specifically infects basal layer keratinocytes, and the virus life cycle is linked to keratinocyte differentiation (3).Normally, keratinocytes present antigen to primed T cells (4–6) However, HPV16 E7 transgenic primary keratinocytes are not susceptible to CTL-mediated lysis by E7-specific T cells *in vitro* (7). This defect of endogenous antigen presentation could be specific to the E7 antigen, as keratinocytes expressing OVA as transgene are sensitive to cell-mediated lysis by CD8^+^ OT-1 cells (5, 6). In addition to *in vitro* studies using immortalized or primary keratinocytes, a transgenic mouse model expressing HPV16E7 protein controlled by a keratin 14 (K14) promoter (K14.E7) has been used to study persisting HPV16E7 gene expression, as this model harbors the molecular features of CIN3 tissue (8). Multiple local factors including suppressive immunity, mediated by CD4^+^CD25^+^ cells against multiple immunogens (9), IFNγ-producing NKT cells (10), impaired antigen processing and T cell activation by antigen-presenting cells (APCs) (11) are observed in the skin of K14.E7 transgenic mice, and the ear skin of K14.E7 mice is not rejected when grafted to immunocompetent recipient mice (12), reflecting the failure of clearance of chronic HPV16 infection in some infected humans.

HPV16 E7 protein interacts with multiple proteins including γ-tubulin, p-600, Retinoblastoma (Rb) protein family, HDAC, E2F6, p21, and IRF1 (13). The interaction between E7 and Rb disrupts normal cell cycle regulation, leading to epithelial hyperproliferation, one of the major pathological phenotypes of patients with HPV associated CIN3. However, it is unclear whether suppressed local immunity is a result of E7-associated hyperplasia or some other consequence of expression of the viral protein. To dissect this question, we utilized transgenic mice expressing the E7 protein and with a mutant Rb that is functional for cell cycle regulation but cannot bind E7 (K14.E7xRb^ΔL/ΔL^) (14). While expressing a comparable level of E7 transcript, the skin of K14.E7xRb^ΔL/ΔL^ mice, whether homozygous or heterozygous for the Rb mutation, was found to closely resemble non-transgenic mouse skin (15, 16). To test whether the local immune suppression observed in K14.E7 transgenic mouse skin was due to hyperproliferation or to some other action of E7 protein, we examined immune function in the skin of K14.E7 and K14.E7xRb^ΔL/ΔL^ mice. However, K14.E7xRb^ΔL/ΔL^ skin grafts were not rejected from naïve or E7 primed recipients, and this was associated with reduced frequency of CD11b^+^ DC, as well as the low expression of maturation markers- CD80 and CD86 on migratory DC subtypes in the skin draining lymph nodes. More importantly, adaptive immune responses to skin-directed antigen in K14.E7xRb^ΔL/ΔL^ mice were comparable to those in non-transgenic wild-type mice, and K14.E7xRb^ΔL/ΔL^ transgenic keratinocytes could present endogenous E7-antigen and be recognized by antigen specific CD8 T cells *in vitro*, unlike E7 transgenic keratinocytes.

## Results

### Disruption of E7-Rb Interaction in E7 Transgenic Mice Restores the Peripheral CD8 T Cell Response to That of Non-transgenic Animals

**Figure 1:**
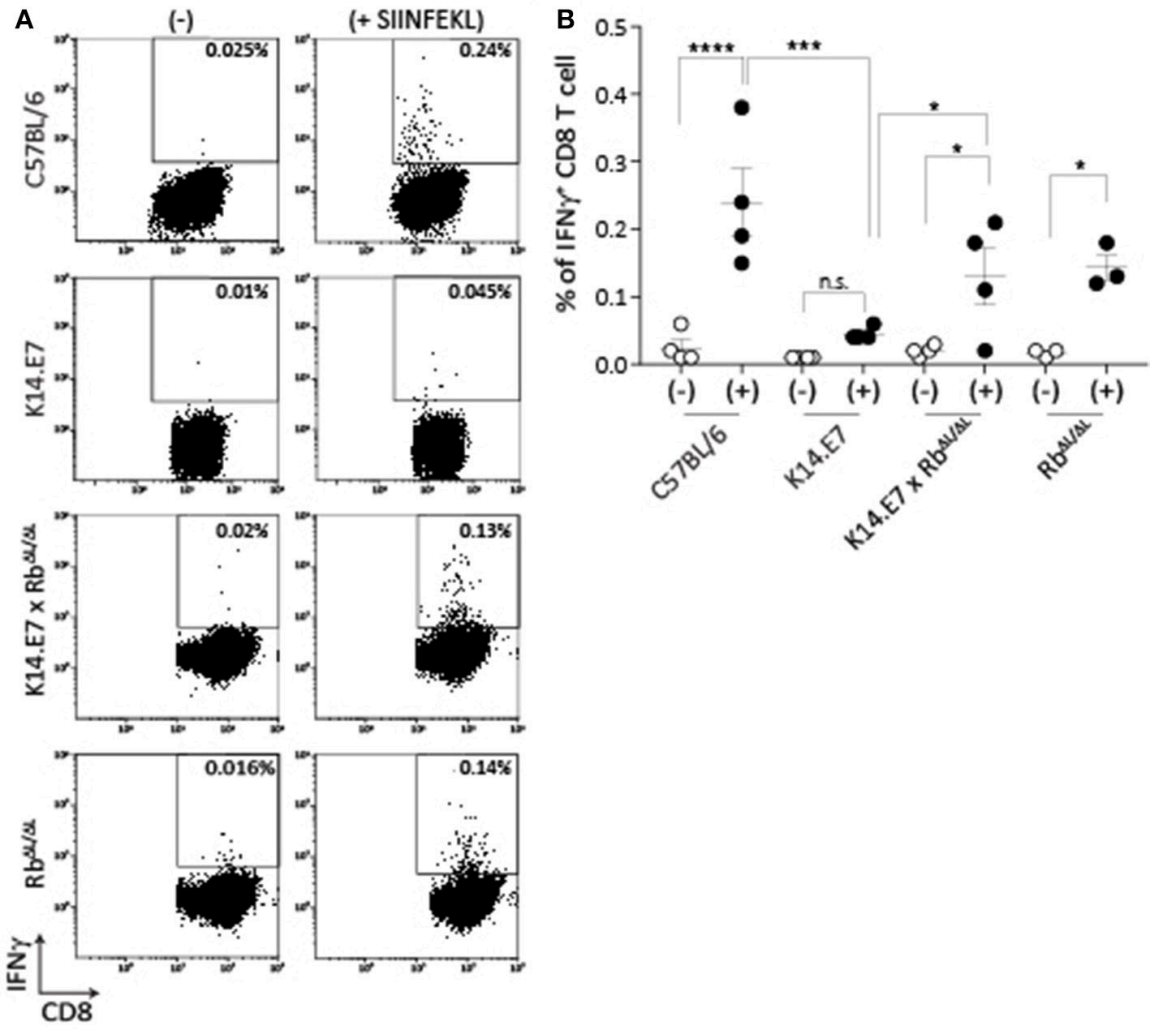
K14.E7xRb ^ΔL/ΔL^ and non-transgenic mice respond equally to intradermal immunization. OVA specific CD8 T cell responses in the draining LN following intradermal immunization were assessed as IFNγ production following *in vitro* peptide re-stimulation. **(A)** Representative FACS plots pre-gated on TCRβ+ CD8 T cells showing IFNγ production with (+) or without (-) SIINFEKL re-stimulation. **(B)** Quantitative result showing mean ± SEM with *n* > 3 for each mouse type. Open circle, without stimulation; closed circle, with stimulation. Analyses were done using one-way ANOVA, with Bonferroni's multiple comparison tests. Result significance was shown, where ^*^p < 0.05, ^***^p < 0.001, and ^****^p < 0.0001.

Mice expressing E7 as a transgene in epithelial cells have altered immune function locally in skin. The major effects of E7 in epithelial cells are mediated via binding to the Rb cell cycle regulatory protein. To determine whether disruption of E7-Rb interaction impacts on the altered immune response in E7 transgenic skin, we first examined induction of peripheral CD8 T cell responses, as these are impaired in E7 transgenic animals to multiple immunogens (9). K14.E7 mice and K14.E7 x Rb^ΔL/ΔL^ mice were immunized with ovalbumin (OVA) and QuilA as adjuvant intradermally, and IFNγ production by CD8 T cells from the draining lymph node in response to SIINFEKL was measured 6 days after immunization. Re-stimulation with SIINFEKL resulted in similar numbers of IFNγ producing CD8 T cells for immunized K14.E7xRb^ΔL/ΔL^ and Rb^ΔL/ΔL^ animals (Figure 1), while K14E7 animals similarly immunized showed significantly lower numbers of IFNγ producing CD8 T cells than C57 animals, as previously demonstrated. Thus, expression of E7 in skin without hyperproliferation does not seem to impair adaptive immune priming.

### K14.E7xRb^ΔL/ΔL^ Skin Is Not Rejected From Immunocompetent Syngeneic Recipients

**Figure 2:**
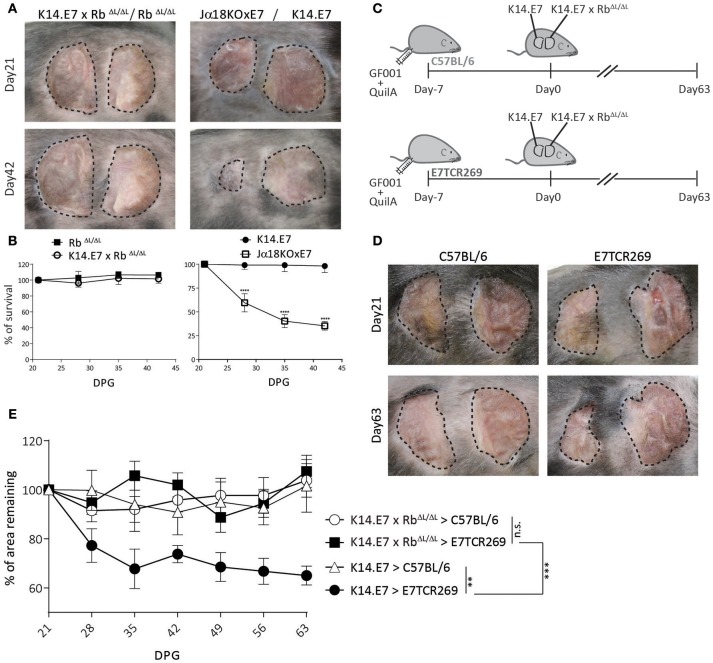
K14.E7xRb^ΔL/ΔL^ skin grafts are not rejected from immunocompetent syngeneic recipients. **(A)** Grafts of donor ear skin, as shown, onto C57BL/6 recipients at 21 and 42-days post grafting. **(B)** Area remaining of graft at the indicated time points (DPG). **(C)** C57BL/6 and E7TCR269 recipients were immunized with GF001 (50 μg) and QuilA (5μg) subcutaneously 7 days before grafting. **(D)** Representative photos of graft-bearing recipients on day 21 and day 63. K14.E7 skin (left) and K14.E7xRb^ΔL/ΔL^ skin (right). **(E)** Area remaining of graft at the indicated time points, compared to the graft on day 21. DPG, day post grafting. Analyses were done using one-way ANOVA, with Bonferroni's multiple comparison tests. Error bars showing mean ± SEM with n≥3. Result significance was shown, where ^**^p < 0.01, ^***^p < 0.001, and ^****^p < 0.0001.

K14.E7xRb^ΔL/ΔL^ mice and non-transgenic mice have similar skin-infiltrating lymphocytes (16), and exhibit similar CD8 T cell responses to immunization (Figure 1), as well as a similar transcriptomic profile to non-transgenic mouse skin (16, 17). We therefore hypothesized that E7-expressing skin lacking hyperplasia might be susceptible to immune mediated rejection. To test this, we grafted K14.E7xRb^ΔL/ΔL^ skin onto syngeneic non-transgenic mice. As controls, we grafted hyperplastic NKT cell deficient Jα18KO × E7 skin, which is susceptible to rejection, (10) and K14.E7 skin, which is not rejected. iNKT deficient E7 skin grafts (Jα18KO × E7) showed rejection, defined as more than 50% shrinkage within 42 days (Figures 2A,B). However, both K14.E7xRb^ΔL/ΔL^ and Rb^ΔL/ΔL^ skin grafts showed no evidence of rejection (Figures 2A,B).

As there is no chemokine mediated accumulation of regulatory T cells in K14.E7xRb^ΔL/ΔL^ skin, as observed in K14.E7 skin, a different mechanism must prevent E7 specific priming or effector functions where E7 transgenic grafts are not associated with hyperplasia. Passive transfer of sufficient E7-specific cytotoxic CD8 T cells enabled rejection of E7-expressing skin grafts following immunization (12). We therefore tested whether a similar approach would lead to rejection of K14.E7xRb^ΔL/ΔL^ skin grafts. The frequency of various APC subtypes in skin, lymph nodes and spleen between E7TCR269 and C57BL/6 did not show significant differences despite increased frequency of T cells expressing Vβ12 receptor in E7TCR269 animals (Supplementary Figure 1). K14.E7 and K14.E7xRb^ΔL/ΔL^ skin together were grafted on to the same E7TCR269 recipient that have an expanded E7-specific CD8 T cell repertoire (9) (Supplementary Figure 1), and to non-transgenic recipients, with prior immunization with an H-2 D^b^ restricted peptide of E7 (GF001) (Figure 2C). Consistent with our previous findings, K14.E7 grafts showed ~40% reduction in size on immunized E7TCR269 recipients (Figures 2D,E). In contrast, K14.E7xRb^ΔL/ΔL^ grafts showed no reduction in size on either C57BL/6 or E7TCR269 recipient mice. These data demonstrate that failure of E7 transgenic skin grafts to prime an E7-specific response is not the sole reason for failure of rejection of K14.E7xRb^ΔL/ΔL^ grafts. As E7-expression levels are similar in K14.E7 and K14.E7xRb^ΔL/ΔL^ skin (16), it is unlikely that the expression level of the antigen influenced the fate of K14.E7xRb^ΔL/ΔL^ skin grafts. Thus, we hypothesized three possibilities of why the K14.E7xRb^ΔL/ΔL^ skin grafts are tolerated- (i) K14.E7xRb^ΔL/ΔL^ skin graft alone may not initiate sufficient skin antigen presenting cell migration and activation in the draining lymph nodes to prime T cells; (ii) K14.E7xRb^ΔL/ΔL^ skin grafts fail to attract effector cells, consistent with their lower expression of T cell attracting chemokines (16); (iii) E7 transgenic KC fail to present antigen effectively in the absence of the local inflammatory signals (IL-1β, IL-17) that are a feature of K14.E7 skin (8).

### Limited Presence of Graft-Derived Dendritic Cells in the Draining Lymph Node Was Observed Upon K14.E7xRbΔ*L*/Δ*L* Skin Grafting

**Figure 3:**
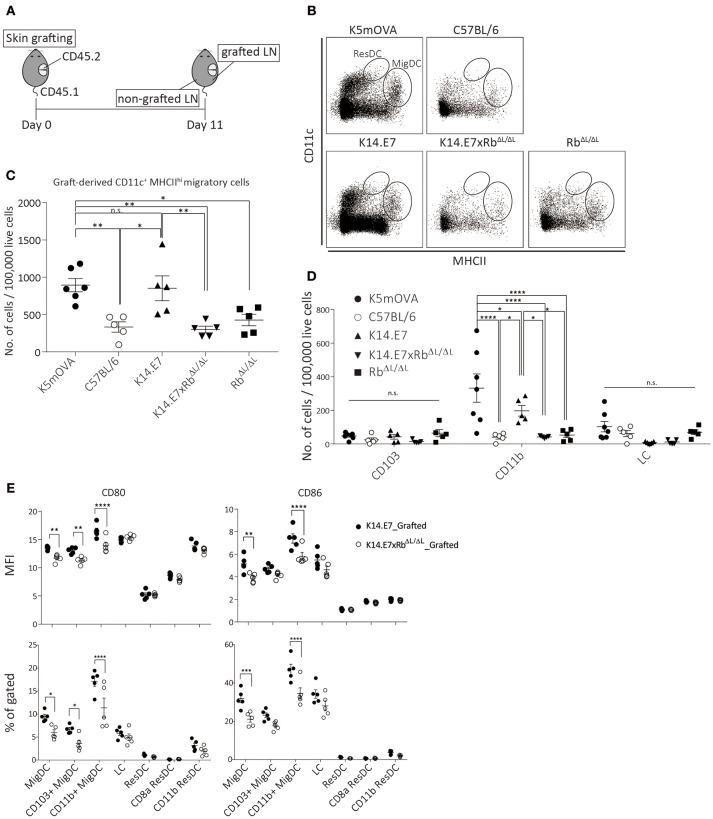
The presence of K14.E7xRb^ΔL/ΔL^ and K14.E7 DCs from grafted skin in the draining lymph node. Skin-derived DC in graft draining and control lymph node of CD45.1 recipients receiving K5mOVA, C57BL/6, K14.E7, K14.E7xRb^ΔL/ΔL^, or Rb^ΔL/ΔL^ (CD45.2) skin grafts. **(A)** experimental outline. Draining lymph nodes (axillary and iguanual) were harvested 11 days after grafting from either grafted side or non-grafted side of one graft recipient. **(B)** representative plots showing graft-derived (CD45.1–) DC under live CD45.1–gate. **(C)** quantitative result of migratory DC [vertical oblong gate in **(B)**] in grafted draining lymph node of recipients receiving indicated type of skin grafts. Plot showing the number of cells per 100,000 live cell analyzed. **(D)** number of cells per 100,000 live cells analyzed of graft-derived MigDC subtypes (CD103+, CD11b+ and LC) from lymph nodes of mice receiving indicated skin grafts. **(E)** MFI (top) and percentage (bottom) of CD80 and CD86 positive cells of various DC subtypes in grafted-drianing lymph nodes receiving either K14.E7 (closed circle) or K14.E7xRb^ΔL/ΔL^ (opened circle) skin graft. All statistics were done with one-way ANOVA with Bonferroni post-test. Plots show mean value with SEM. Result significance was shown, where ^*^p < 0.05, ^**^p < 0.01, ^***^p < 0.001, and ^****^p < 0.0001.

To further investigate the induction and attraction of effector T cells to K14.E7xRb^ΔL/ΔL^ skin grafts, we firstly determined whether K14.E7xRb^ΔL/ΔL^ skin-derived dendritic cells, that might present antigen to activate effector T cells and induce local chemo attraction and inflammation, could be found in the graft draining lymph node. K14.E7xRb^ΔL/ΔL^ skin was transplanted onto congenic (CD45.1) recipients and draining lymph nodes (axillary and inguinal) from both grafted and non-grafted flanks were harvested 11 days post grafting, after resolution of any grafting induced inflammation. When K14.E7 skin was similarly transplanted, the frequency of graft derived APC in the skin graft dropped significantly 7–10 days post grafting and remained low till day 14. Thus, 10–14 days post grafting was expected to be when graft-derived DC would be found in the draining lymph node (Supplementary Figure 2A). To determine appropriate gating for separation of CD45.1 from CD45.2 lymphocytes, naïve lymph nodes from C57BL/6, B6.SJL.Ptprca, and 1:1 mixture of the two were stained with CD45.1 antibody (Supplementary Figure 2B). C57BL/6 skin grafts were used to control for effects of grafting, Rb^ΔL/ΔL^ grafts were used as control for effects of Rb mutation and K5mOVA transgenic skin grafts, which are effectively rejected (18, 19) (Figure 3A) provided a positive control. Graft recipient CD11c^+^ cells were similar in number in the draining lymph node, and in the contralateral node, regardless of graft type (Supplementary Figures 3A,B). Higher number of graft-derived CD11c^+^ cells were found in the graft draining lymph node than in the contralateral node (Supplementary Figure 3C). However, less graft-derived (CD45.1^−^) CD11c^+^ cells were found in graft draining lymph nodes of animals receiving C57BL/6, K14.E7xRb^ΔL/ΔL^, or Rb^ΔL/ΔL^ grafts when compared with K5mOVA or K14.E7 skin grafts (Supplementary Figures 3C,D). Graft-derived cells in the draining nodes (CD45.1^−^) were further analyzed based on CD11c and MHCII expression level, to differentiate migratory DC (CD11c^+^ MCHII^hi^ migDC) from resident DC (CD11c^+^ MCHII^int^ ResDC). A significant number of MigDC, but not ResDC, was found in the draining lymph nodes (Figure 3B), confirming that the majority of donor derived lymph node cells were of graft origin. The frequency of migratory DC (CD11c^+^ MCHII^hi^) in the lymph nodes of animals receiving K14.E7 grafts was similar to those in K5mOVA skin graft recipients (Figure 3C). Frequencies of MigDC in the draining lymph nodes of animals receiving K14.E7xRb^ΔL/ΔL^ skin grafts were however significantly lower than for K5mOVA grafts and K14.E7 grafts (Figure 3C). Examining the different DC subtypes, the number of graft-derived CD11b^+^ DC per 100,000 live cells in lymph nodes, but not CD103^+^ DC or LC, was significantly different amongst groups receiving different skin grafts (Figure 3D). Thus, these data confirm that there are more K5mOVA and K14.E7-derived CD11b^+^ DC than K14.E7xRb^ΔL/ΔL^ and Rb^ΔL/ΔL^-derived DC.

Next, we further examined the maturation state of different subtypes of DC in the draining lymph nodes derived from either K14.E7 or K14.E7xRb^ΔL/ΔL^ skin, based on the expression of CD80 and CD86. Analyzed at the same time point, the percentage and MFI of CD80 and CD86 on migratory DC, including CD103^+^ and CD11b^+^ migratory DC, are significantly higher on cells derived from the K14.E7 skin graft (Figure 3E). No significant difference was observed in resident DC subtypes. This suggests that K14.E7xRb^ΔL/ΔL^ skin graft-derived DC not only cannot sufficiently migrate to the draining lymph node, but also that the migrated DC are not mature DC. Together, these results show that less antigen presenting cells migrate from grafted K14.E7xRb^ΔL/ΔL^ skin than from K14.E7 skin, which is consistent with failure of effective presentation of graft derived antigen, and absence of local induction of T cell derived pro-inflammatory cytokines.

### Keratinocytes With Disrupted E7-Rb Interaction Engage With Antigen-Specific CD8 T Cells and Enable CTL-Mediated Lysis

**Figure 4:**
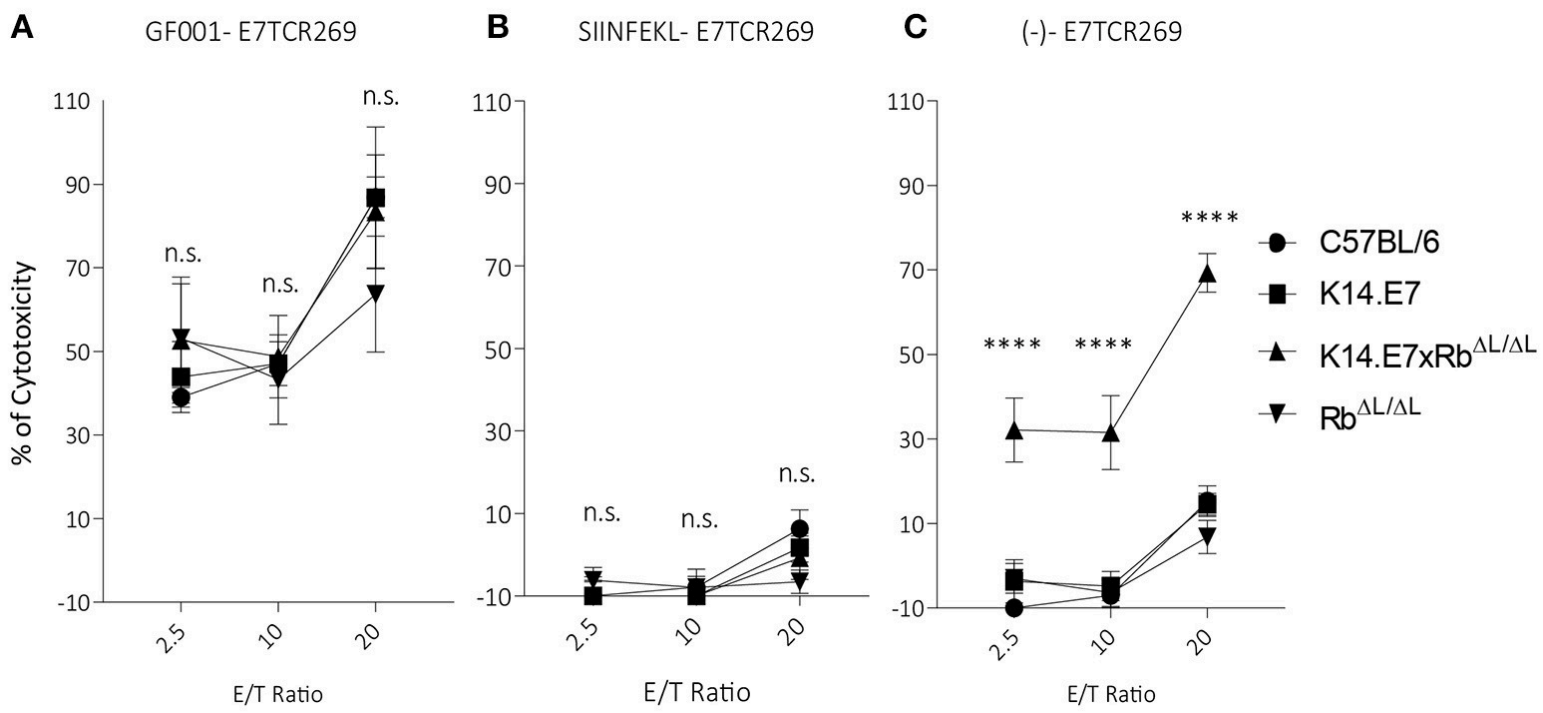
Keratinocytes from K14.E7xRb ^ΔL/ΔL^ skin present endogenous antigen and are susceptible to CTL-mediated lysis. Keratinocytes isolated from the epidermis of C57BL/6, K14.E7, K14.E7xRb^ΔL/ΔL^, and Rb^ΔL/ΔL^ mice were pulsed with **(A)** GF001 peptide **(B)** SIINFEKL peptide or **(C)** without peptide (-) and co-cultured with E7-specific effector T cells (E7TCR269) at the shown effector/target (E/T) ratios. Keratinocyte death was assessed as LDH release above background from no-peptide pulsed KC and effector T cell alone. Two independent experiments were done. Target cell maximum release and spontaneous release of LDH are plotted in Supplementary Figure 4. Data analyzed using two-way ANOVA and applied with Bonferroni post-test. Error bars showing mean ± SEM with 3 to 5 biological replicates in each experimental group. Result significance was shown, where ^****^p < 0.0001.

Previously, we have demonstrated that activated antigen-specific CD8 T cells can enter both hyperplastic (K14.E7) and normoplastic (K14.E7xRb^ΔL/ΔL^) E7-expressing epidermis but only a very low number of antigen-specific T cells are retained in the K14.E7xRb^ΔL/ΔL^ epidermis (20). Graft rejection requires effector T cells to engage antigen expressing keratinocytes. To test whether K14.E7 and K14.E7xRb^ΔL/ΔL^ keratinocytes are equally effective at presenting endogenous E7 antigenic peptides to effector T cells, flow cytometry sorted keratinocytes were exposed to HPV16E7-peptide (GF001) (Figure 4A), OVA (SIINFEKL) (Figure 4B) or not exposed (Figure 4C), and were co-cultured with E7 peptide-specific effector T cells, or with SIINFEKL-specific OT-1 cells (Supplementary Figures 5A–C) as controls. Consistent with our previous findings, keratinocytes from K14.E7 epithelium were susceptible to CTL-mediated lysis only when provided with exogenous E7 peptide antigen (6) (Figure 4A). In contrast, keratinocytes of the K14.E7xRb^ΔL/ΔL^ skin were susceptible to lysis by E7 peptide specific T cells without exogenous peptide provision (Figure 4C). These results demonstrate that E7-Rb interaction or E7-induced cell proliferation interferes with effective MHC class I antigen presentation, and that disruption of E7-Rb interaction or of the resulting cellular proliferation can restore the susceptibility of E7-expressing keratinocytes to cytotoxic T cell killing. Failure of rejection of K14.E7xRb^ΔL/ΔL^ skin must therefore reflect failure of retention and activation of skin effector T cells in the E7 transgenic skin, perhaps a consequence of failure of local T helper cell activation.

## Discussion

In this study, we modeled the induced immune response to a tumor antigen expressed in premalignant epithelial cells, using HPV16 E7 protein transgenic animals. We have previously shown that immune responses are impaired following intradermal immunization in HPV16E7-expressing hyperproliferative epithelium (21). Here, we show that this impairment is at least in part a consequence of the skin epithelial hyperplasia associated with E7-Rb interaction, as effective CD8 T cell responses could be produced by intradermal OVA immunization in K14.E7xRb^ΔL/ΔL^ mice lacking epithelial hyperproliferation.

HPV16E7 transgenic skin models cervical intraepithelial neoplasia at a transcriptional level (8), and is associated with suppressed local immunity, as extensively studied by our laboratory and others (10–12, 15, 22, 23). Local immunoregulatory mechanisms associated with E7-expressing hyperproliferative skin have been shown previously to inhibit rejection of K14.E7 skin grafts. One of the mechanisms contributing to the suppressive immunity is altered cytokine and chemokine production favoring a regulatory/inhibitory response in hyperproliferative epithelium (16). Paradoxically, given the impaired immune response associated with intradermal immunization of K14.E7 transgenic animals, we observed in the current study that grafts of hyperproliferative E7 transgenic skin placed on immunocompetent animals primed to E7 were more effectively rejected than grafts of normally proliferating K14.E7xRb^ΔL/ΔL^ skin. This disparity may however in part reflect the altered immune repertoire associated with E7 expression and epithelial proliferation in the developing thymus of the K14.E7 transgenic animal (24) as well as the higher number of lymphocyte recruitment by the hyperproliferative graft (20). It is worth noting that in the previous report (20), effector T cell recruitment to K14.E7 and K14.E7xRb^ΔL/ΔL^ skin graft was assessed 3 days post grafting and showed the lack of effector T cell recruitment in K14.E7xRb^ΔL/ΔL^ skin but not in K14.E7 skin. In this manuscript, we further assessed the outcome of the skin grafts and agreed with previous finding, in which the lack of effector T cell recruitment can be one of the causes of graft tolerance. In studies conducted by Broom et al. (25) and Hadis et al. (19) in our lab, grafts of skin from mice expressing OVA from a keratin 5 promoter are rejected when transplanted. In a study using K14 ovalbumin transgenic mice, the authors observed no significant inflammatory reaction after adoptive transfer of activated OVA specific OT-1 cells (26, 27). However, in studies conducted by Shibaki et al. inflammatory reaction could be triggered by transferring OT-1 cells without further activation (28). In each of these studies, the observations were made on transgenic animals, rather than on grafted transgenic skin as in our OVA studies.

Rejection of an antigen-expressing skin transplant is typically initiated by migration of graft-derived DC to the draining lymph node, where they activate host T cells (29, 30). In addition, antigen processing and presentation by the recipient's antigen presenting cells results in an influx of recipient CD4, CD8 T cells, and CD11b^+^ macrophages to the graft and graft rejection (31). Rejection requires presentation of antigen to the recruited recipient effector cells to elicit antigen specific cytotoxic effect (32, 33). The hypothesis relates to the migration of potentially regulatory APC from K14.E7x Rb^ΔL/ΔL^ grafts, when compared with K14.E7 grafts which promote APC migration and suppress systemic E7 immune responses (11). Such migration is not seen with C57 grafts, or with the appropriate negative control (Rb^ΔL/ΔL^). In our model, graft-derived APC migration to the draining lymph node was less with K14.E7xRb^ΔL/ΔL^ grafts than with K14.E7 grafts, and this was not simply due to the thickness of skin graft, as frequency of APC migrating from K5mOVA skin grafts, which is known to be rejected upon transplantation, of similar thickness was also greater. We note that at the time point of observation, the skin-derived DC in the draining lymph node may include cells that directly migrated from the graft and also the cells that proliferated after migration. Furthermore, the lower frequency of migratory CD11b^+^ DC derived from K14.E7xRb^ΔL/ΔL^ skin was not due to the thickness of skin graft, as both K5mOVA and K14.E7xRb^ΔL/ΔL^ skin are normoplastic, having similar numbers of total live CD45^−^ cells, and similar frequency of various APC subtypes in the skin (Supplementary Figures 3E,F). Increased migration might reflect the more inflammatory environment in hyperproliferative K14.E7 skin, that would enhance antigen processing and presentation of endogenous antigen by KC (34). More effective lymphocyte recruitment by the hyperproliferative K14.E7 than by the normal proliferative K14.E7 graft (20) might also facilitate graft rejection, and impaired recruitment is a consequence of lesser production of chemokines (16).

Noting that few effector T cells are recruited to K14.E7xRb^ΔL/ΔL^ skin, we hypothesized that if sufficient antigen-specific effector T cells were present, antigen-bearing keratinocytes from the K14.E7xRb^ΔL/ΔL^ skin would be susceptible to T cell mediated killing, and tested this *in vitro*. We observed that K14.E7 keratinocytes were susceptible to lysis only if exogenous peptide was provided, but keratinocytes from K14.E7xRb^ΔL/ΔL^ animals were also able to present endogenous antigen resulting in lysis. This was not due to differences in transcription of E7 protein between the two cell populations (16). Keratinocytes from hyperproliferative epithelium thus appear to be less effective *in vitro* at processing or presentation of endogenous E7 antigen for presentation by MHC Class I. Their better rejection *in vivo* might thus reflect better MHC class I presentation of antigen by keratinocytes in an inflammatory environment. Determining whether helper T cell mediated enhancement of E7 presentation enhances E7 graft rejection is the focus of ongoing studies.

Recently, a small thiadiazolidinedione molecule binding to pRb through the L × C × E motif has been identified which selectively interferes with HPV16E7-Rb but not HPV18E7-Rb interaction (35). This small molecule interrupts the E7-Rb interaction in the same manner as K14.E7xRb^ΔL/ΔL^. Moreover, this molecule demonstrated effects on cell cycle and reduced TC-1 tumor volume after treatment in immune incompetent mice. While drug mediated interruption of E7- Rb interactions might slow E7 dependent tumor growth and facilitate induction of effective peripheral immunity to E7, our results suggest that this alone may not be sufficient to clear E7 infected cells. However, a combination of a small molecule inhibitor of Rb-E7 interaction, and more effective antigen presentation through immunization, might help to eliminate E7 infected tissues.

## Materials and Methods

### Mice

All mice were maintained in Translational Research Institute (TRI) Biological Research Facility (BRF) under specific pathogen free conditions. For experimental work, female mice were used at 8–12 week of age. C57BL/6 and B6.SJL.Ptprca mice were obtained from Animal Resources Centre (Perth, Australia) and OT-1 mice were purchased from The Jackson Laboratories (Bar Harbor, ME, USA). K14.E7 (36) and K5mOVA (18) mice were maintained at the TRI-BRF. Heterozygous Rb^ΔL/ΔL^ transgenic and NKT cell deficient Jα18^−/−^ mice (37) were obtained from Dr. Fred Dick and Mark Smyth (Melbourne, Australia), respectively, and maintained locally at TRI-BRF. K14.E7xRb^ΔL/ΔL^ and Jα18^−/−^xE7 mice were generated by mating heterozygous K14.E7 with heterozygous Rb^ΔL/ΔL^ and homozygous Jα18^−/−^, respectively. K14.E7xRb^ΔL/ΔL^ mice have a single allele of E7 transgene and at least one allele of mutated Rb. OT-I mice were purchased from The Jackson Laboratories (Bar Harbor, ME, USA). E7TCR269 mice were bred as previously described (9).

### Immunization

Immunizations were performed by injecting 5 μg QuilA (Sigma-Aldrich, St. Louis, MO, USA) with 50 μg OVA (A5503, Sigma-Aldrich, St. Louis, MO, USA) or E7 peptide-GF001 (RAHYNIVTF, synthesized by Auspep Pty Ltd, (Melbourne, Australia), with purity >80%) in 20 μl PBS into one ear pinnae intradermally or 200 μl PBS subcutaneously.

### Isolation of Cells From Lymph Node and Spleen

The isolation was done as previously described (16). Lymph nodes were digested with 1 μg/μl, of collagenase D (Roche) and 0.2 μg/μl of DNase, (Roche) for 30 min at 37°C prior to passing through the cell strainer.

### FACS, Antibodies and Reagents

FACS analysis was undertaken as previously described (10). Foxp3 Fix/Perm Concentrate and Diluent kit (eBioscience, San Diego, USA) was used for intracellular staining of IFNγ. Anti-mouse monoclonal antibodies to CD45 (30-F11), CD45.1 (A20), CD8α (53-6.7), TCRβ (H57-597), CD4 (GK1.5), EpCAM (G8.8), I-A/I-E (MHCII) (M5/114.15.2), CD103 (M290), CD11c (HL3), CD11b (M1/70), IFNγ (XMG1.2) and the corresponding isotype antibodies were purchased from Biolegend (San Diego, USA), eBioscience (San Diego, USA), BD Bioscience (San Jose, USA).

### *In vitro* Cytotoxic T Cell Assay

Effector cells (E) were harvested from the spleen of E7TCR269 and OT-1 mice 6 days after GF001 and OVA immunization, respectively. Splenocytes were cultured in standard RPMI and supplemented with IL-2 (1 U/mL, Peprotech, NJ, USA) overnight and CD8 T cells were enriched using biotinylated antibodies (16). Target keratinocytes (T) were sorted using BD FACSAria Fusion Sorter and pulsed with 10 μg/mL of either GF001 or SIINFEKL (Sigma-Aldrich, St. Louis, MO, USA) peptide. Effector and target cells were co-cultured at 20:1, 10:1, or 2.5:1 ratio for 5 h and cell-mediated cytotoxicity assay was performed using CytoTox 96® Non-Radioactive Cytotoxicity Assay kit (Promega, Madison, WI, USA) following manufacturer's instruction. Percentage of cytotoxicity was calculated as per manufacturer's instruction (each value calculated has been subtracted with medium background):

(1) $$\%\;of\;Cytotoxicity=\frac{\left(mean\;read\right)-\left(effector\;spontaenous\;release\right)-(KC\;spontaneous\;release)}{\left(KC\;maximum\;release)-(KC\;spontaneous\;release\right)}\;x\;100\%$$

### Skin Grafting

Skin grafting was performed as previously described (38). Briefly, skin graft recipients were shaved on the flank and shaved skin was cut out in the size that matches the donor skin. Ears from the donor mice were split into dorsal and ventral part and grafted onto recipient mice. Bactigras (Smith and Nephew, Australia) was placed, following by bandaging. The bandage was taken off 7 days after the procedure. Day 21 post-grafting was set as baseline for comparison as the skin grafts are well-healed and no local inflammation was observed.

### Statistical Analysis

Prism 7 (GraphPad Software, La Jolla, CA) software was used for statistical analysis and to prepare the plots. Unless otherwise stated, all analyses were done using one-way ANOVA, with Bonferroni's multiple comparison tests. Two-way ANOVA with Bonferroni's post-test analysis was used in Figure 4. All plots show mean value with SEM. Paired-student *t*-test was used in Figure 3B. Result significance was shown, where ^*^p < 0.05, ^**^p < 0.01, ^***^p < 0.001, and ^****^p < 0.0001.

## Ethics Statement

All animal experiments and procedures were performed in compliance with the ethical guidelines of the National Health and Medical Research Council of Australia and approved by the University of Queensland Animal Ethics Committee (UQDI/367/13/NHMRC and UQDI/452/16).

## Author Contributions

PK, IF, GL and SM conceived the study. PK and ST conducted the experiments. PK wrote the manuscript. ZT and all other authors analyzed the results and reviewed the manuscript.

### Conflict of interest statement

The authors declare that the research was conducted in the absence of any commercial or financial relationships that could be construed as a potential conflict of interest.
