# Supplementary material for: Recruitment of Antigen Presenting Cells to Skin Draining Lymph Node From HPV16E7-Expressing Skin Requires E7-Rb Interaction
Source: Front Immunol. 2018 Dec 18;9:2896. doi: 10.3389/fimmu.2018.02896 (PMC6305623; doi:10.3389/fimmu.2018.02896)
Supplement: Supplementary file 1 [file Data_Sheet_1.docx]

Supplementary Material

Recruitment of APC to Skin Draining Lymph Node from HPV16E7-Expressing Skin Requires E7-Rb Interaction

Paula Kuo, Siok Min Teoh, Zewen K. Tuong, Ian H. Frazer*, Graham R. Leggatt, Stephen R. Mattarollo

*** Correspondence:**Ian Frazer, The University of Queensland, Faculty of Medicine, The University of Queensland Diamantina Institute, Translational Research Institute
i.frazer@uq.edu.au

# Supplementary Figures


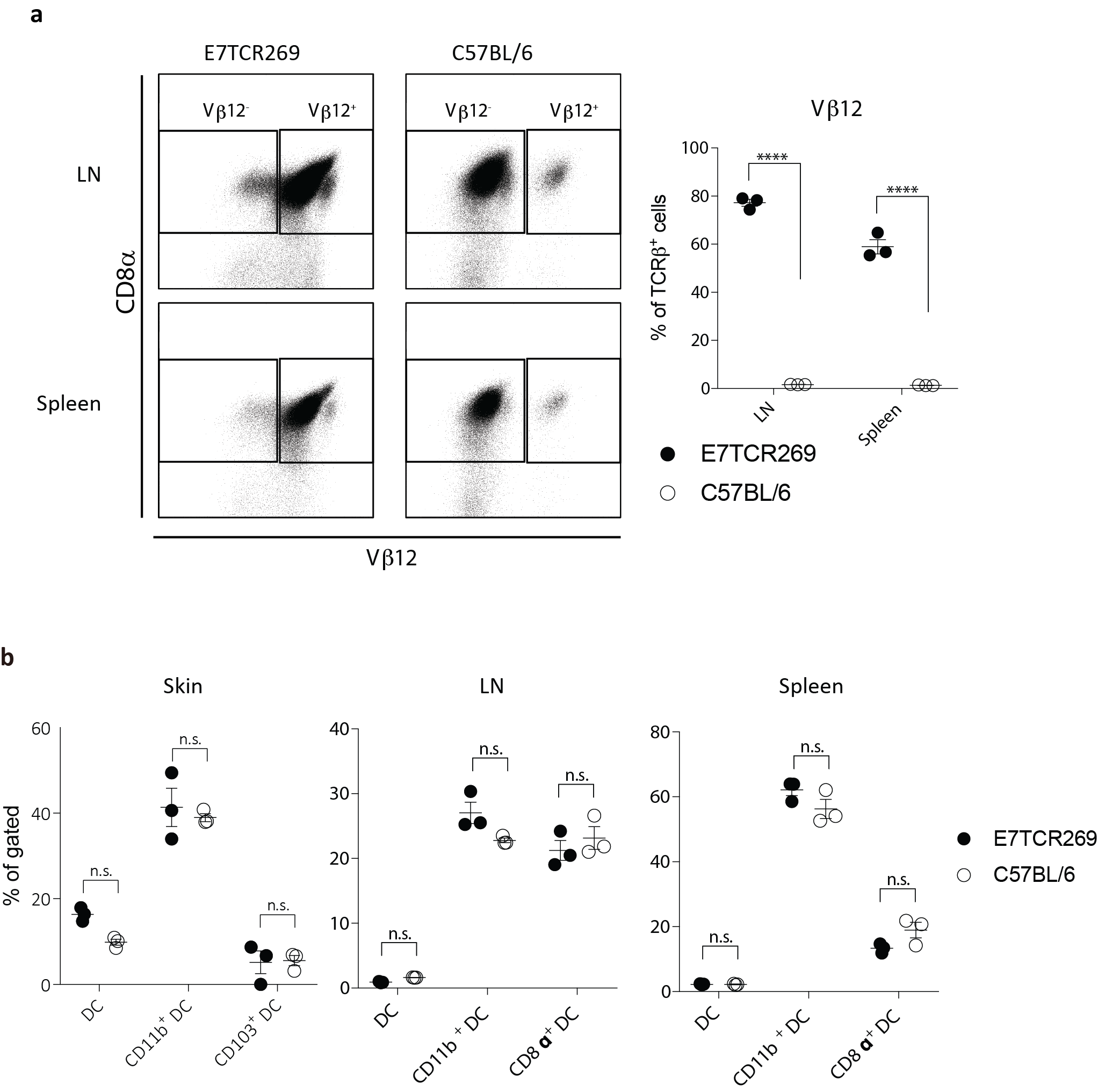


**Supplementary Figure 1** Frequency of T cells with receptor Vβ12 in lymph node and spleen of E7TCR269 and C57BL/6 on day 0 of skin grafting experiment, corresponding to Figure 2c. Plots are pre-gated on live TCRβ^+^ T cells. Statistics were done with matched two-way ANOVA with Bonferroni post-test. Result significance was shown, where ****p<0.0001.


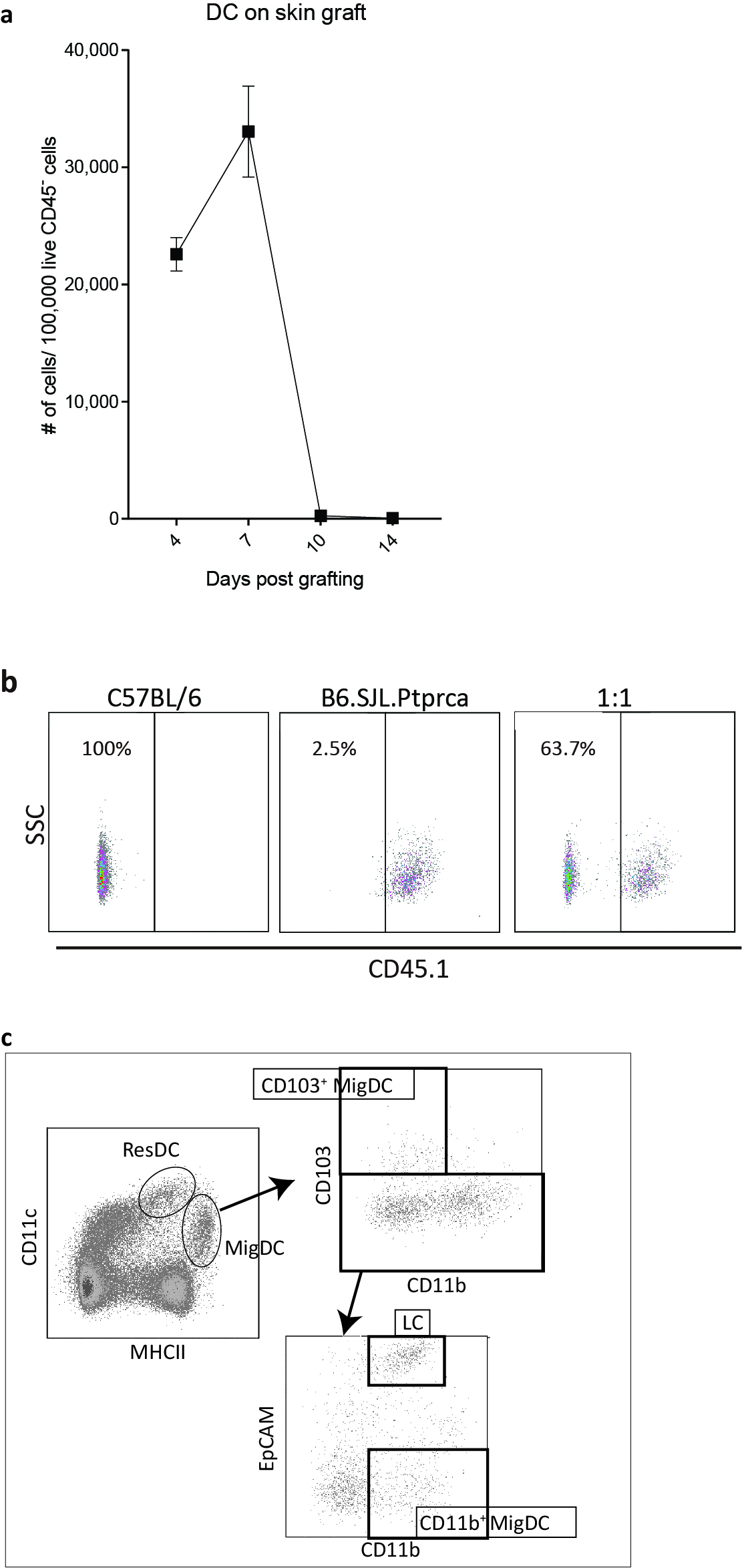


**Supplementary Figure 2.** (a) Number of DC on K14.E7 skin graft on 4, 7, 10 and 14 days post skin grafting. Plot shows mean value of five skin grafts on different recipients with SEM. (b) Naïve lymph node from C57BL/6, B6.SJL.Ptprca and 1:1 mixture stained with CD45.1 antibody to establish gating. Plots were pregated on live CD11c^+^ cells. (c) gating strategy of resident DC (ResDC), migratory DC (migDC) and DC subtypes. Representative plots from one recipient-derived live cell gate were shown.


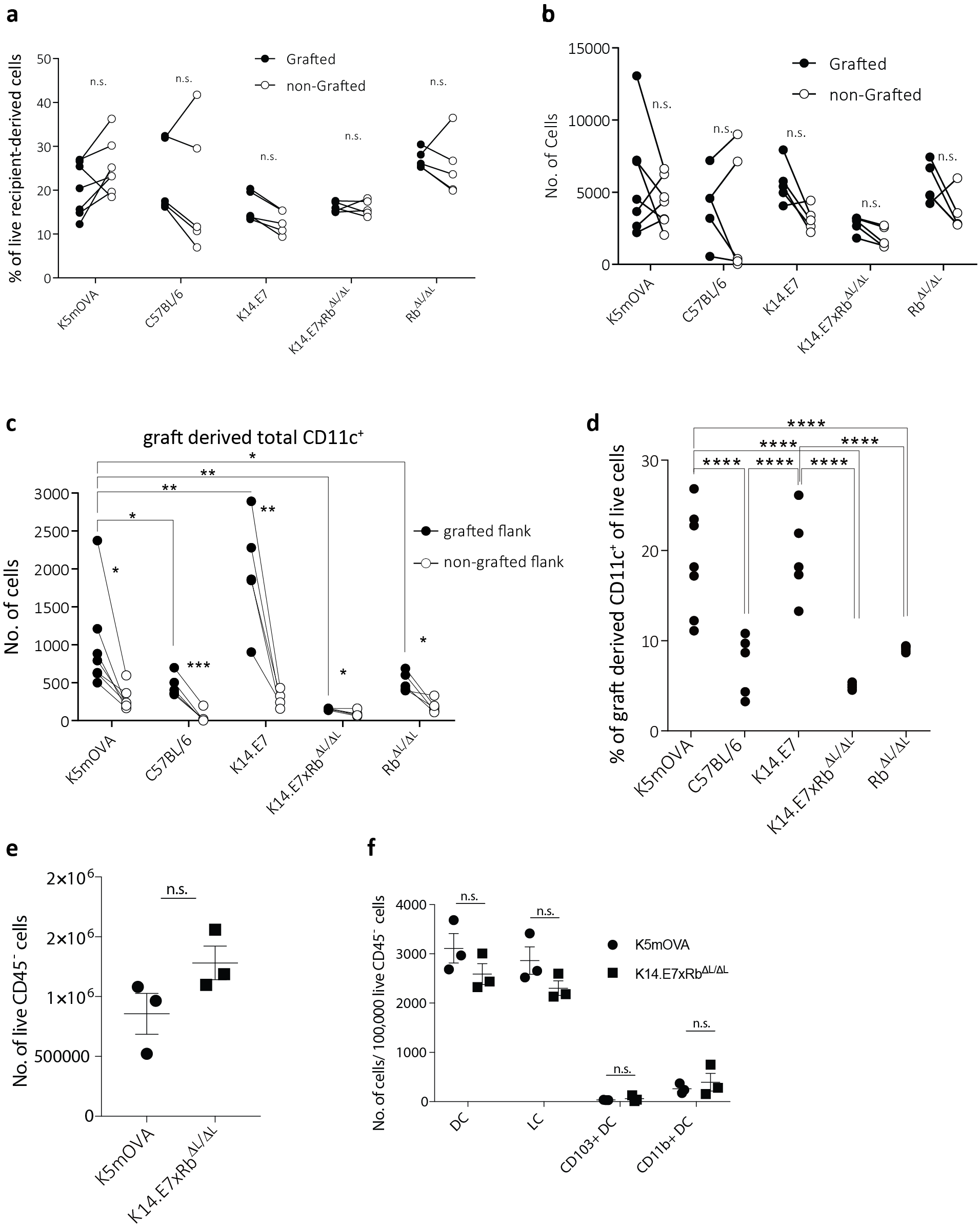


**Supplementary Figure 3.** Percentage (a) and numbers (b) of recipient-derived CD11c^+^ cells under live CD45.1^+^ gate. Closed and open circle showing draining lymph nodes analyzed from the grafted and non-grafted site, respectively. n.s., non-significant. (c) total numbers of graft-derived cells (CD45.1^-^), pre-gated on live CD11c^+^ cells, in lymph nodes of grafted and non-grafted side. Paired student t-test applied for comparison between grafted and non-grafted flanks. Matched two-way ANOVA with Bonferroni post-test applied for comparison among grafted flanks receiving different skin grafts. (d) percentages of graft-derived live CD11c^+^ cells in graft draining lymph nodes receiving indicated skin grafts. (e) number of live CD45^-^ cells in K5mOVA and K14.E7xRb^ΔL/ΔL^ skin. Un-paired student t-test was used for analysis. (f) number of APC subsets per 100,000 live CD45^-^ cells in skin of K5mOVA and K14.E7xRb^ΔL/ΔL^. If not already mentioned, statistics were done with one-way ANOVA with Bonferroni post-test. (c and d) result significance was shown, where *p<0.05, **p<0.01, ***p<0.001 and ****p<0.0001.


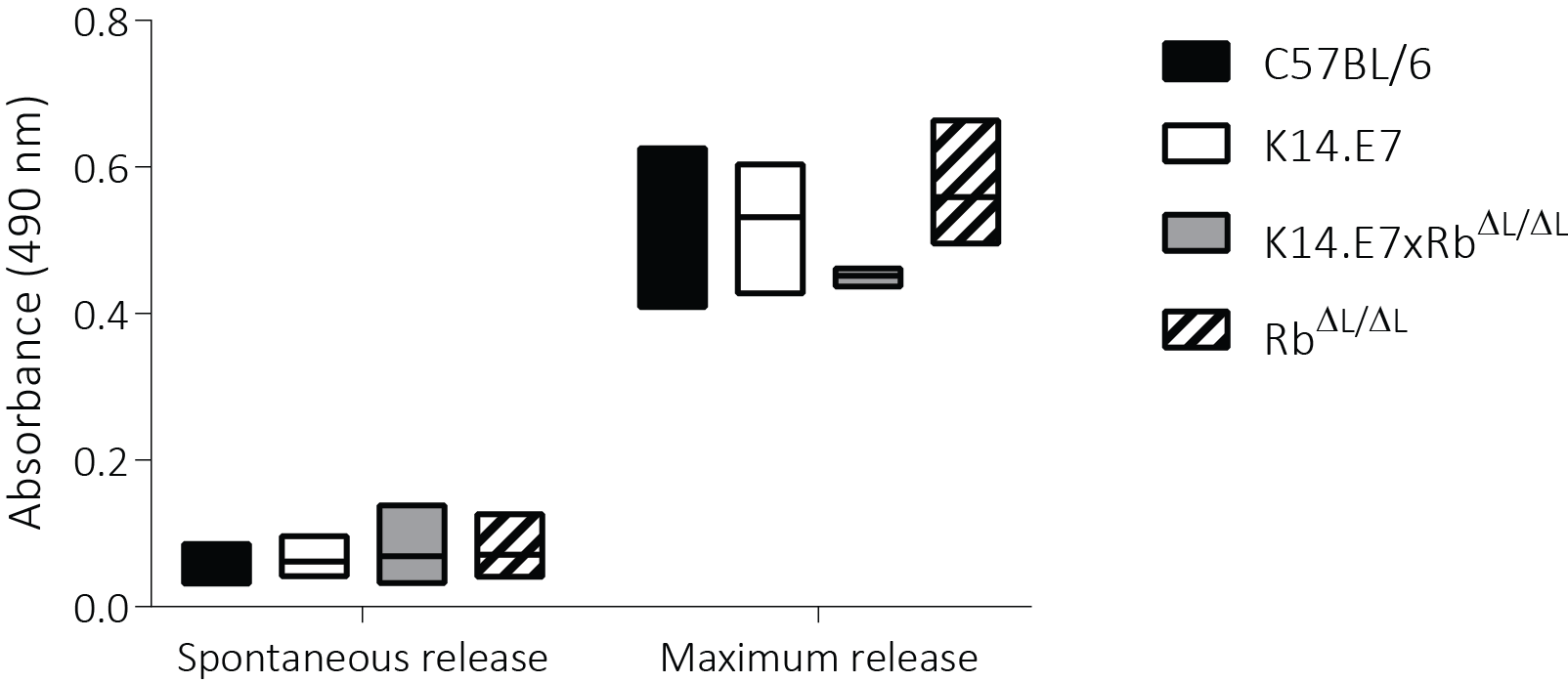


**Supplementary Figure 4.** Target spontaneous and maximum LDH release at the same target cell number in Figure 4 Bar graph showing minimum and maximum absorbance at 490nm of three biological replicates.


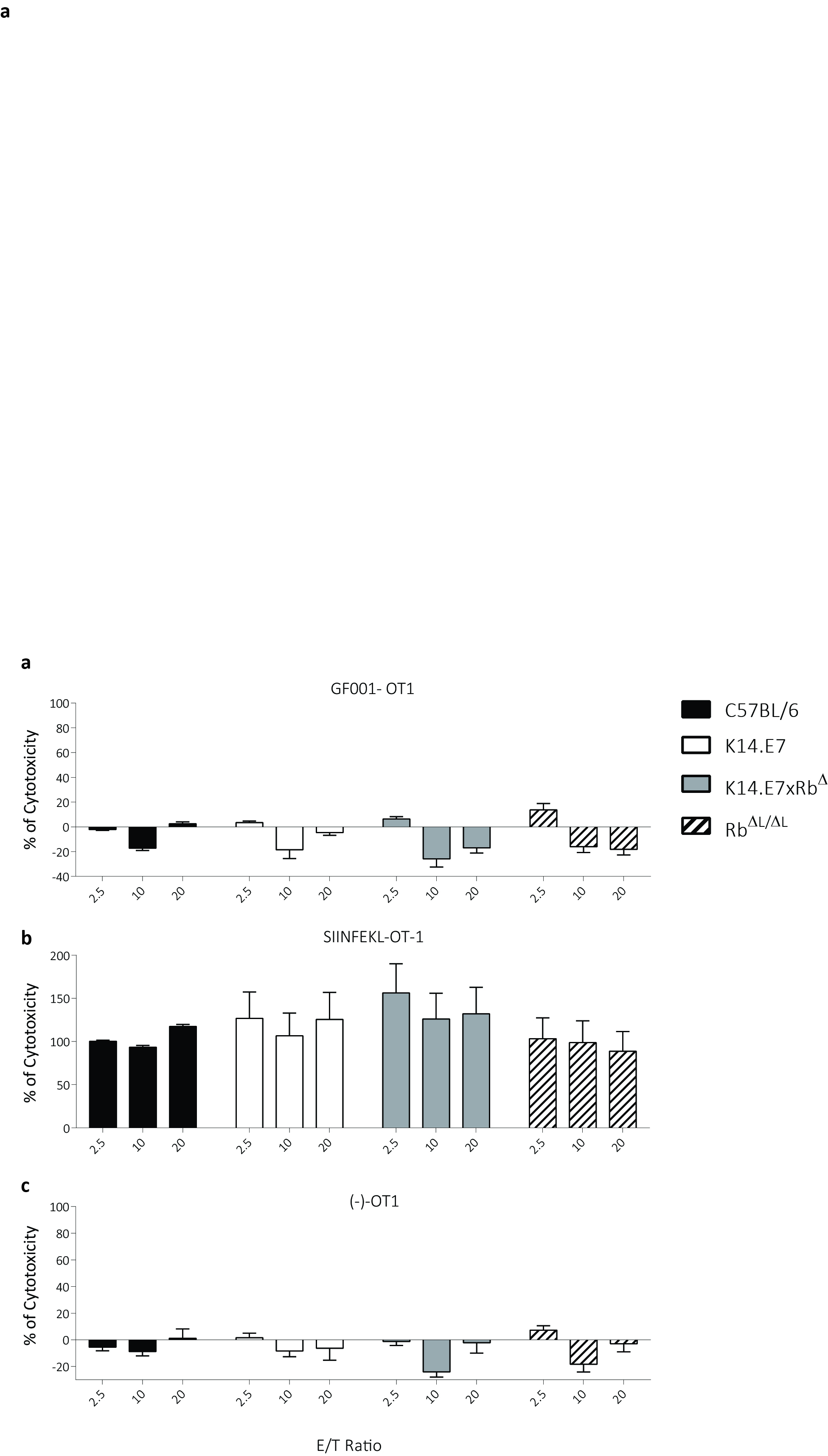


**Supplementary Figure 5.** Control groups of antigen specific keratinocyte *in vitro* killing. Keratinocytes isolated from the epidermis of C57BL/6, K14.E7, K14.E7xRb^ΔL/ΔL^, and Rb^ΔL/ΔL^ mice were pulsed with GF001, SIINFEKL peptide or no peptide (-) and co-cultured with SIINFEKL-specific effector T cells (OT-1) at the shown effector/target (E/T) ratios. Cytotoxicity is calculated with respect to keratinocyte death, which is assessed by LDH release using the following equation. Each value is subtracted with medium control.

$$\% of Cytotoxicity=\frac{\left( mean read \right)-\left( effector spontaenous release \right)-(KC spontaneous release)}{(KC maximum release)-(KC spontaneous release)} x 100\%$$
